# Characteristics of self-injurious behaviour and early traumatic experiences: associations with emotional reactivity, depression and aggression in university students

**DOI:** 10.1192/bjo.2024.862

**Published:** 2025-03-11

**Authors:** Irina Jarvers, Elisa Heidingsfelder, Angelika Ecker, Stephanie Kandsperger, Romuald Brunner, Daniel Schleicher

**Affiliations:** Department of Child and Adolescent Psychiatry and Psychotherapy, University of Regensburg, Regensburg, Germany

**Keywords:** Aggression, depressive disorders, emotional reactivity, self-harm, trauma and stressor-related disorders

## Abstract

**Background:**

A lifetime history of non-suicidal self-injury (NSSI) is a risk factor for subsequent behavioural and emotional problems, including depression, aggression and heightened emotional reactivity. Traumatic experiences, which are frequently reported by individuals with NSSI, also show predictive links to these mental health problems. However, the exact connections between these areas and their subdomains remain unclear.

**Aims:**

To explore in detail the relationships of specific characteristics of NSSI (e.g. termination in adolescence, duration, frequency, reinforcement mechanisms) and various types of traumatic experience (emotional, physical, sexual) with distinct aspects of emotional reactivity (sensitivity, intensity, persistence), aggression (behavioural, cognitive, affective) and severity of depression in university students.

**Method:**

Via online survey, 150 university students aged 18 to 25 years, who had self-injured at least once, provided information on NSSI, and completed questionnaires including the Childhood Trauma Questionnaire, Patient Health Questionnaire, Emotion Reactivity Scale, and Aggression Questionnaire. Regression analyses were conducted to determine risk factors linked to increased depression scores, aggression and emotional reactivity. The study was pre-registered in the German Clinical Trials Register (DRKS00023731).

**Results:**

Childhood emotional abuse contributed to emotional reactivity, aggression and depressive symptom severity (*β* = 0.33–0.51). Risk factors for sustained NSSI beyond adolescence included increased automatic positive reinforcement (odds ratio: 2.24).

**Conclusions:**

Childhood emotional abuse significantly contributes to emotional and behavioural problems and needs to be considered in NSSI therapy. NSSI was found to persist into adulthood when used as an emotion regulation strategy.

Non-suicidal self-injury (NSSI) refers to self-inflicted damage to one’s own body tissue without suicidal intent.^1^ NSSI is most common in adolescence, with high prevalence rates of 7.50% to 46.50% and an average first occurrence between 12 and 14 years of age, followed by lower prevalence (4.00–23.00%) in adulthood.^2^ However, an NSSI prevalence of 38.90% has also been observed among university students.^2,3^ Therefore, NSSI is not only found in clinical samples but also in the general population and in university students. Across studies, women were more likely to report a lifetime history of NSSI; however, these gender differences were greater in clinical samples.^4^ The intention behind NSSI is usually to resolve intrapersonal difficulties such as negative emotions and cognitive states; this represents a key reinforcement mechanism that maintains this behaviour.^5^ Whereas ‘automatic’ intrapersonal functions (i.e. emotion regulation) are most frequently reported by people with NSSI, ‘social’ interpersonal functions (i.e. communication of stress and strengthening of social support) are less common.^6^ Overall, NSSI in adolescence is considered to be a strong risk factor for various mental health difficulties including emotional and behavioural problems in young adulthood.^7^ Here, it may be important to consider the correlations among emotional reactivity, psychopathology and self-injurious behaviours.^8–11^ For example, NSSI has historically been considered to be a strong predictor of depressive symptom severity.^12^ Although NSSI excludes suicidal intentions, it is associated with an increased risk of suicide.^13^ There are also indications that people who injure themselves have increased pain tolerance, although this aspect has often been neglected, and correlations with psychological constructs are unclear.^14^ Furthermore, people who show self-injurious behaviours can be seen as a risk group for externalising behaviours such as aggression.^15^ Traumatic experiences, including emotional and physical abuse or neglect, are also frequently reported by people with NSSI.^16^ Importantly, negative experiences in childhood and adolescence show strong correlations with maladaptive behaviours and mental health problems later in life,^17–20^ including depression,^21,22^ increased emotional reactivity^8,23^ and externalising symptoms in the aggressive–impulsive spectrum.^24–26^ In summary, NSSI^7^ and traumatic experiences^17–20^ are risk factors for mental health problems and maladaptive behaviours in young adulthood. Overall associations have already been shown between NSSI and other phenomena, including emotional reactivity, aggression and depressive states.^8,12,15^ However, it is largely unclear which characteristics of NSSI (e.g. frequency, reinforcement mechanisms) or which types of early traumatic experience (e.g. emotional versus physical versus sexual) are linked to which emotional or behavioural symptoms in adulthood (e.g. depression, aggression and emotional reactivity). Further studies of the subdomains of emotional reactivity (intensity, sensitivity and persistence of emotional reactions)^8^ and aggression (affective, cognitive or behavioural with verbal and physical aggression)^27^ are also lacking. As some individuals engage in NSSI solely during adolescence, whereas others engage in NSSI continuously into adulthood,^28^ studies should examine more closely which exact NSSI characteristics and which types of trauma have a central role in the continuation of NSSI. Although high-frequency repetitive patterns of NSSI^28^ and negative life events in general^29^ appear to be important risk factors, less attention has been paid to reinforcement mechanisms. There are initial indications that NSSI is more likely to be continued if it serves intrapersonal functions.^30^ By contrast, previous mental health treatment and use of NSSI for interpersonal functioning seem more likely to be associated with cessation of NSSI.^30^

Therefore, the present study examined whether and to what extent NSSI characteristics and early traumatic experiences are risk factors for the development of emotional and behavioural problems in university students. More precisely, we analysed the effects of different self-injury characteristics regarding duration, frequency, reinforcement mechanisms (social as well as automatic-negative and automatic-positive reinforcement), suicidal tendencies, gender, former therapy and pain perception, as well as early traumatic events (emotional, physical and sexual traumas), on current emotional reactivity (emotional sensitivity, arousal and persistence), aggression (anger, hostility and/or distrust, physical and verbal aggression) and depression symptom severity in young adulthood. We hypothesised that NSSI would form part of a vicious cycle, in which heightened emotional reactivity, aggression and depressive symptoms are associated with engaging in NSSI. In turn, despite NSSI serving as a brief and maladaptive emotion regulation strategy, its characteristics would exacerbate emotional reactivity, aggression and depressive symptoms, perpetuating the cycle. In addition, we examined which risk factors contributed to continuous engagement in NSSI compared with NSSI only in adolescence. We hypothesised that use of NSSI as a maladaptive emotion regulation strategy would be associated with continuously engaging in NSSI from adolescence to adulthood.

## Method

### Study design and participants

An *a priori* power analysis for the cross-sectional online study was conducted using G*Power^31^ based on an effect size of Cohen’s *d* = 0.59 (corresponding to *f* = 0.30) from a meta-analysis of the relationship between NSSI and impulsivity^32^ as a core factor for aggression and emotional reactivity. Based on a power of 80%, this resulted in a sample size of at least *N* = 90. Recruitment was carried out via notices, flyers, mailing lists and internet advertisements among the general student population. The online study was conducted via the PsyToolkit platform.^33,34^ Inclusion criteria were: being a university student between the ages of 18 and 25 years, with a sufficient understanding of the German language and a history of NSSI. The survey was open from April to September 2021. At the end of the online survey, participants had the opportunity to take part in a voluntary prize draw for 25 gift vouchers worth 15€ each. For this purpose, they were forwarded a link that was independent of the survey to enter their e-mail address.

A total of *N* = 240 students participated in the online survey. Of these, 23 were excluded as they did not fill in any information after beginning the survey. A further two were excluded as they claimed to have never engaged in NSSI, 64 were excluded owing to missing questionnaire data, and, finally, one individual was excluded as they were the only person identifying as gender-diverse, resulting in a final sample of *n* = 150. For group comparisons, the ten individuals who had begun engaging in NSSI during adulthood (after 18 years of age) were excluded, as they could not be meaningfully compared with the 43 students engaging in NSSI solely in adolescence (before 18 years of age) or the 97 engaging in NSSI continuously (before and after 18 years of age, but not necessarily at the time of the survey).

### Procedure and measures

Digital informed consent to participate was obtained from all students after the inclusion criteria and main objectives of the study had been explained via the study information at the beginning of the online survey. Trigger warnings and contact information for counselling centres and study staff were displayed at regular intervals. In addition, participation in the survey was voluntary and could be terminated at any time without giving reasons by closing the browser window.

The authors assert that all procedures contributing to this work comply with the ethical standards of the relevant national and institutional committees on human experimentation and with the Helsinki Declaration of 1975, as revised in 2013. All procedures involving human subjects and/or patients were approved by the Ethics Committee of the University of Regensburg (reference number: 20-2041-101). Furthermore, this study was pre-registered in the German Clinical Trials Register (DRKS00023731).

When participants had provided consent, demographic data regarding gender (female, male, diverse), age, study programme and field of study were first requested. Subsequently, key characteristics of NSSI were surveyed, including course (e. g. ‘Have you deliberately injured yourself as a child or adolescent (0–17 years)?’, ‘Have you deliberately injured yourself since the age of 18?’; ‘yes’ or ‘no’), duration (e.g. ‘How many years have you been doing this?’; 1: ‘up to 1 year’, 4: ‘6 years or longer’), frequency (‘Please try to remember the year you most often self-injured as a child or teenager and/or as an adult. How often did you do this in that year?’; 1: ‘1 time’, 4: ‘more than 5 times’), suicide attempt (e.g. ‘Have you attempted suicide in your life?’; ‘yes’ or ‘no’) and psychiatric treatment (e.g. ‘Have you ever received psychiatric, psychotherapeutic or psychological treatment for this behaviour?’; ‘yes’ or ‘no’).

Subsequently, pain perception (‘Did you experience pain during self-harm?’; 0: ‘no pain’, 3: ‘severe pain’) and 22 different reasons for NSSI (0: ‘never’, 3: ‘often’) were queried about using parts of a German version of the Functional Assessment of Self-Mutilation (FASM).^35,36^ Four scales of reinforcement mechanisms can be calculated on the basis of these 22 functions: automatic-negative reinforcement (e.g. ‘to stop bad feelings’), automatic-positive reinforcement (e.g. ‘to feel relaxed’), social-negative reinforcement (e.g. ‘to avoid being with people’) and social-positive reinforcement (e.g. ‘to get control of a situation’).^37^ The analysis of psychometric properties of the FASM showed acceptable values with regard to concurrent validity and internal consistency.^38–40^ In the present sample, the FASM had an internal consistency (Cronbach’s *α*) of 0.85.

Next, individuals’ experiences of their current emotional reactivity were recorded using the 21 items (0: ‘not at all like me’, 4: ‘completely like me’) of a German version of the Emotion Reactivity Scale (ERS).^8,11^ The following subscales were calculated: sensitivity (e.g. ‘My feelings get hurt easily.’), arousal/intensity (e.g. ‘I experience emotions very strongly.’) and persistence (e.g. ‘When I feel emotional, it’s hard for me to imagine feeling any other way.’). The individual scales exhibited strong internal consistencies and demonstrated significant convergent, divergent and criterion validities for behavioural inhibition/activation and various psychopathologies.^8^ In the present sample, the ERS had an internal consistency (Cronbach’s *α*) of 0.94.

The various dimensions of aggression in current adulthood were measured using the German version of the Aggression Questionnaire (29 items, 1: ‘does not apply’, 4: ‘strongly agree’).^27,41^ The questionnaire records two behavioural tendencies, physical aggression (e.g. ‘I get into fights a little more than the average person.’) and verbal aggression (e.g. ‘I often find myself disagreeing with people.’); one affective component, anger (e.g. ‘I have trouble controlling my temper.’); and one cognitive component, hostility and/or distrust (e.g. ‘I am sometimes eaten up with jealousy.’). In addition to acceptable values for the internal consistency and test–retest reliability of the questionnaire, the differential validity was supported.^41^ In the present sample, the Aggression Questionnaire had an internal consistency (Cronbach’s *α*) of 0.88.

The nine items (0: ‘not at all’, 3: ‘nearly every day’) of the German version of the Brief Patient Health Questionnaire (PHQ-9)^42^ were used to screen for current depressive symptoms (past 2 weeks). Psychometric studies showed high internal consistency, very good test–retest reliabilities, and confirmation of construct and criterion validity.^43^ The PHQ-9 scores can be categorised into no to minimal depression (scores 0–4), mild depression (scores 5–9), moderate depression (scores 10–14) and moderately severe depression (scores 15–19).^42^ In the present sample, the PHQ-9 had an internal consistency (Cronbach’s *α*) of 0.89.

At the end of the online survey, participants were asked to reflect on traumatic experiences during their childhood and adolescence (up to the age of 18 years) using the German short version of the Childhood Trauma Questionnaire (CTQ).^44–46^ The questionnaire consists of 28 items (1: ‘not at all’, 5: ‘very frequently’) and covers the following areas: physical abuse, emotional abuse, sexual abuse, physical neglect and emotional neglect. The prevalence of traumatic experiences was computed by considering all participants that scored above 13 points on emotional abuse, ten points on physical abuse, eight points on sexual abuse, 15 points on emotional neglect and ten points on physical neglect.^46^ The construct validity, internal consistencies and model fit of the five-factor structure could be confirmed as sufficient; however, it is important to critically assess the physical neglect scale.^46^ In the present sample, the CTQ had an internal consistency (Cronbach’s *α*) of 0.80.

### Statistical analysis

Descriptive statistics, correlations and multivariable regression models were computed using SPSS Statistics (Version 28.0, for Windows; https://www.ibm.com/products/spss-statistics). As a first step, chi-squared and Mann–Whitney *U* tests were used to compare groups of students who engaged in NSSI solely during adolescence with those who continued NSSI into adulthood, with a focus on differences in traumatic experiences, emotional reactivity, aggression and depressive symptoms between the groups.

As a second step, linear multivariable regressions were computed to identify significant predictors of students’ emotional reactivity, aggression and depressive symptoms. Predictors included gender, areas of traumatic experiences in childhood (CTQ), duration of NSSI, frequency of NSSI, pain perception (FASM), presence of a suicide attempt, reinforcement mechanisms of NSSI (FASM) and whether participants had received therapy for NSSI. For emotional reactivity and aggression, additional linear regressions were conducted, focusing on the respective subscales.

In addition, we examined whether the aforementioned variables could predict whether an individual engaged solely in NSSI during adolescence or continued this behaviour into adulthood using a binominal logistic regression. The false discovery rate (FDR)^47^ was used to correct for multiple comparisons, and the *P*-value threshold was set to 0.05.

## Results

**Fig. 1:**
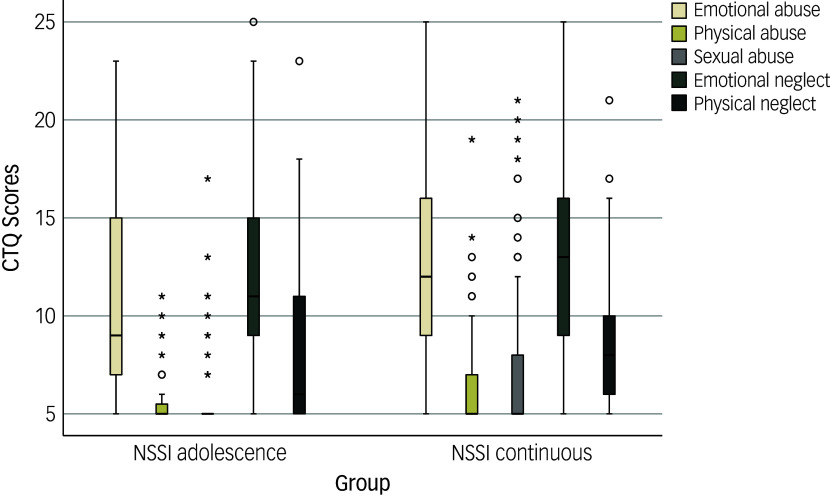
Distribution of Childhood Trauma Questionnaire (CTQ) scores across subscales and groups. NSSI, non-suicidal self-injury. Circles represent mild outliers (1.5 × interquartile range below first quartile or above third quartile); asterisks represent extreme outliers (3.0 × interquartile range below first quartile or above third quartile).

Among the *n* = 150 students engaging in NSSI at some point in their lives, 84.00% (*n* = 126) were female, and 54.70% (*n* = 82) were currently studying within a bachelors’ programme, 15.30% (*n* = 23) within a masters’ programme and 30.00% (*n* = 45) for a state exam (degree from German universities or state institutions for subjects in the fields of medicine and law). Regarding study programmes, 16.70% (*n* = 24) were studying to be teachers, 16.00% (*n* = 23) were studying natural sciences, 11.10% (*n* = 16) social sciences, 11.10% (*n* = 16) psychology, 10.40% (*n* = 15) medicine, 6.30% (*n* = 9) economics, 5.60% (*n* = 8) law, 3.50% (*n* = 5) engineering and 19.40% (*n* = 28) other subjects. A total of 13.30% (*n* = 20) had engaged in NSSI within the past week, 14.00% (*n* = 21) within the past month, 24.00% (*n* = 36) within the past year, 22.00% (*n* = 33) 2–3 years ago, 12.70% (*n* = 19) 4–5 years ago and 14.00% (*n* = 21) 6 or more years ago. See Table 1 for an overview of questionnaire scores for the whole sample and group comparisons between participants solely engaging in NSSI during adolescence and those engaging in NSSI continuously (before and after the age of 18 years). After FDR correction, there were no significant differences between the two NSSI groups. The distribution of CTQ scores across the groups is depicted in Fig. 1.

**Table 1:** Descriptive sample characteristics and group comparisons

Variables	Total sample (*n* = 150), %	NSSI adolescence (*n* = 43), %	NSSI continuous (*n* = 97), %	Group comparisons
Gender	84.00	88.40	83.50	*χ* ^2^ = 0.55, *P* = 0.457
Suicide attempts	18.00	4.65	24.74	*χ* ^2^ = 7.95, *P* = 0.005*
Prior therapy	36.00	25.58	38.14	*χ* ^2^ = 2.09, *P* = 0.149

	Mean or median (s.d. or range)	Mean or median (s.d. or range)	Mean or median (s.d. or range)	Group comparisons
**Age**	22.00 (18.00–25.00)	21.00 (19.00–25.00)	22.00 (18.00–25.00)	*z* = −1.37, *P* = 0.00171
**CTQ**	46.72 (14.01)	44.32 (13.15)	48.74 (14.35)	z = −1.73, *P* = 0.00083
Emotional abuse	12.17 (5.30)	11.42 (5.52)	12.81 (5.27)	*z* = −1.64, *P* = 0.00101
Physical abuse	6.27 (2.44)	5.72 (1.50)	6.62 (2.79)	*z* = −2.02, *P* = 0.00043
Sexual abuse	7.13 (3.92)	6.21 (2.63)	7.76 (4.41)	*z* = −2.40, *P* = 0.00016
Emotional neglect	12.89 (5.03)	12.40 (4.99)	13.31 (5.02)	*z* = −1.02, *P* = 0.00307
Physical neglect	8.40 (3.35)	8.58 (4.10)	8.47 (3.08)	*z* = −0.57, *P* = 0.00566
**ERS**	43.49 (17.44)	40.70 (16.34)	44.22 (17.71)	*z* = −1.13, *P* = 0.00260
Sensitivity	20.90 (8.50)	19.93 (8.19)	21.16 (8.65)	*z* = −0.87, *P* = 0.00385
Intensity	14.21 (6.70)	12.56 (6.17)	14.62 (6.60)	*z* = −1.77, *P* = 0.00077
Persistence	8.38 (3.67)	8.21 (3.75)	8.44 (3.70)	*z* = −0.50, *P* = 0.00618
**Aggression Questionnaire**	56.05 (11.91)	53.98 (10.93)	57.07 (12.62)	*z* = −0.94, *P* = 0.00346
Physical aggression	13.44 (4.18)	12.49 (2.91)	13.93 (4.74)	*z* = −1.24, *P* = 0.00215
Verbal aggression	10.23 (2.73)	10.02 (2.46)	10.50 (2.86)	*z* = −0.72, *P* = 0.00473
Anger	13.87 (3.91)	13.30 (3.22)	13.98 (4.01)	*z* = −0.61, *P* = 0.00540
Hostility and/or distrust	18.50 (5.17)	18.16 (5.68)	18.66 (5.14)	*z* = −0.34, *P* = 0.00733
**PHQ-9**	12.75 (6.61)	10.56 (6.66)	13.59 (6.61)	*z* = −2.53, *P* = 0.00011*
**FASM**	1.41 (0.68)	1.42 (0.59)	1.39 (0.71)	*z* = −0.58, *P* = 0.00565
**NSSI characteristics**				
NSSI duration	2.64 (1.10)	1.84 (0.79)	3.09 (0.98)	*z* = −6.28, *P* < 0.00001*
NSSI frequency on average^a^	3.66 (0.62)	3.35 (0.81)	3.39 (0.67)	*z* = −0.14, *P* < 0.00889
NSSI frequency in adolescence^a^	3.59 (0.74)	3.35 (0.81)	3.70 (0.68)	*z* = −3.25*, P* < 0.00001*
NSSI frequency in adulthood^a^			3.09 (1.03)	
Reinforcement mechanisms				
Automatic negative	1.97 (0.90)	1.74 (1.02)	2.09 (0.82)	*z* = −1.73, *P* = 0.00084
Automatic positive	1.71 (0.79)	1.36 (0.75)	1.87 (0.76)	*z* = −3.61, *P* < 0.00001*
Social negative	0.23 (0.40)	0.27 (0.46)	0.22 (0.38)	*z* = −0.39, *P* = 0.00696
Social positive	0.52 (0.45)	0.63 (0.56)	0.49 (0.40)	*z* = −0.76, *P* = 0.00446

CTQ, Childhood Trauma Questionnaire; ERS, Emotion Reactivity Scale; FASM, Functional Assessment of Self-Mutilation; NSSI, non-suicidal self-injury; PHQ-9, Brief Patient Health Questionnaire.

a. In the worst year. Group differences significant after FDR correction are marked with an asterisk. NSSI adolescence refers to students engaging in NSSI only during adolescence, and NSSI continuous refers to students who engaged in NSSI continuously from adolescence into adulthood.

Participants who engaged in NSSI continuously (from adolescence into adulthood) had longer duration and frequency of NSSI during adolescence (*P* < 0.001), were more likely to use NSSI for automatic positive reinforcement (*P* < 0.001) and had higher depressive symptomatology (*P* = 0.011) and more frequent suicide attempts (*P* = 0.005) compared with participants that engaged in NSSI solely during adolescence (Table 1). There was no significant difference in the time of first self-injury between the two groups (NSSI adolescence: *M*_age_ = 13.60, s.d._age_ = 2.62; NSSI continuous: *M*_age_ = 14.77, s.d._age_ = 3.64; *U* = 1921.50, *z* = −0.54, *P* = 0.591).

### Linear regressions predicting emotional reactivity (ERS)

To identify variables that contributed to increased emotional reactivity, a multivariable linear regression was computed with the ERS total score as the dependent variable and gender, traumatic experiences in childhood, duration of NSSI, frequency of NSSI, pain perception, presence of a suicide attempt, reinforcement mechanisms of NSSI and whether students had received therapy for NSSI as independent variables (Table 2). The regression model was significant (*F*(12,134) = 3.07, *P* < 0.001) and explained 25.60% of variance in the ERS total score. Significant predictors of higher ERS values after FDR correction were longer duration of NSSI (*β* = 0.20, *P* = 0.003) and more emotional abuse (*β* = 0.33, *P* = 0.003). Regarding the subscales, prior therapy and emotional abuse were significantly associated with the arousal and intensity scale. For the sensitivity and persistence scales, no significant predictors emerged. See Supplementary Table 1 available at https://doi.org/10.1192/bjo.2024.862 for an overview of regressions regarding the ERS subscales.

**Table 2:** Overview of linear regressions predicting emotional reactivity (ERS), aggression (Aggression Questionnaire) and depression (PHQ-9)^a^

Dependent variable	Predictors	***B***	s.e.	**95% CI (** ***B*** **)**	β	***t***	***P***	***R*** **^2^**
ERS	Gender^b^	2.34	4.06	−5.69 – 10.37	0.05	0.58	0.565	0.26
	Prior therapy	6.77	3.03	0.77 – 0.41	0.19	2.23	0.027	
	**NSSI duration**	**3.21**	**1.41**	**0.37 – 1.82**	**0.20**	**2.27**	**0.003**	
	NSSI frequency	−3.89	2.61	−9.06 – 1.28	−0.14	−1.50	0.139	
	Pain perception	−0.36	1.99	−4.29 – 3.57	−0.01	−1.8	0.855	
	**Emotional abuse**	**1.09**	**0.37**	**0.37 – 1.82**	**0.33**	**2.98**	**0.003**	
	Physical abuse	−1.27	0.70	−2.62 – 0.17	−0.17	−1.74	0.084	
	Sexual abuse	−0.53	0.38	−1.29 – 0.22	−0.12	−1.39	0.166	
	Emotional neglect	−0.33	0.40	−1.12 – 0.46	−0.09	−0.82	0.414	
	Physical neglect	−0.49	0.55	−1.58 – 0.60	−0.09	−0.89	0.376	
	Suicide attempt	5.54	3.98	−2.34 – 13.41	0.12	1.39	0.167	
	ANR	0.01	2.16	−4.26 – 4.29	0.00	.01	0.996	
	APR	3.27	2.40	−1.47 – 8.01	0.15	1.37	0.175	
	SNR	4.19	3.47	−2.67 – 11.05	0.10	1.21	0.229	
	SPR	4.11	3.24	−2.30 – 10.62	0.11	1.27	0.207	
Aggression Questionnaire	**Gender** ^b^	**−7.59**	**2.45**	**−12.45 – −2.74**	**−0.23**	**−3.09**	**0.002**	0.42
	Prior therapy	1.31	1.83	−2.31 – 4.94	0.05	0.72	0.475	
	NSSI duration	0.39	0.86	−1.30 – 2.08	0.04	0.46	0.650	
	NSSI frequency	2.19	1.58	−0.93 – 5.31	0.11	1.39	0.168	
	Pain perception	0.22	1.20	−2.15 – 2.59	0.01	0.18	0.855	
	**Emotional abuse**	**1.14**	**0.22**	**0.71 – 1.58**	**0.51**	**5.16**	**<0.001**	
	Physical abuse	−0.35	0.43	−1.19 – 0.49	−0.07	−0.82	0.412	
	Sexual abuse	−0.25	0.23	−0.71 – 0.20	−0.08	−1.09	0.277	
	Emotional neglect	−0.27	0.24	−0.75 – 0.21	−0.12	−1.13	0.263	
	Physical neglect	0.42	0.33	−0.24 – 1.08	0.12	1.27	0.208	
	Suicide attempt	4.28	2.41	−0.48 – 9.03	0.14	1.78	0.078	
	ANR	0.39	1.31	−2.19 – 2.98	0.03	0.30	0.764	
	APR	−1.02	1.45	−3.89 – 1.84	−0.07	−0.71	0.481	
	SNR	3.98	2.10	−0.16 – 8.13	0.13	1.90	0.060	
	SPR	2.56	2.00	−1.31 – 6.44	0.10	1.31	0.193	
PHQ-9	Gender^b^	0.61	1.50	−2.36 – 3.57	0.03	0.40	0.687	0.29
	Prior therapy	1.17	1.12	−1.04 – 3.39	0.09	1.05	0.297	
	NSSI duration	0.41	0.52	−0.62 – 1.45	0.07	0.79	0.429	
	NSSI frequency	−0.91	0.96	−2.81 – 1.00	−0.09	−0.94	0.349	
	Pain perception	0.01	0.73	−1.35 – 1.55	0.01	0.14	0.892	
	**Emotional abuse**	**0.39**	**0.14**	**0.12 – 0.66**	**0.41**	**2.88**	**0.005**	
	Physical abuse	−0.09	0.26	−0.60 – 0.42	−0.03	−0.35	0.728	
	Sexual abuse	−0.17	0.14	−0.45 – 0.11	−0.10	−1.21	0.230	
	Emotional neglect	−0.21	0.15	−0.50 – 0.08	−0.16	−1.42	0.157	
	Physical neglect	0.11	0.20	−0.30 – 0.51	0.05	0.52	0.604	
	Suicide attempt	2.74	1.47	−0.16 – 5.65	0.16	1.87	0.064	
	ANR	0.81	0.80	−0.77 – 2.38	0.11	1.01	0.314	
	APR	1.79	0.88	0.04 – 3.54	0.21	2.03	0.045	
	SNR	1.99	1.28	−0.55 – 4.52	0.12	1.55	0.123	
	SPR	−0.89	1.20	−3.25 – 1.48	−0.06	−0.74	0.459	

ANR, automatic negative reinforcement; APR, automatic positive reinforcement; ERS, Emotion Reactivity Scale; NSSI, non-suicidal self-injury; PHQ-9, Brief Patient Health Questionnaire; SNR, social negative reinforcement; SPR, social positive reinforcement.

a. Significant predictors after FDR correction are marked in bold.

b. Female was coded as 1 and male as 0.

### Linear regressions predicting aggression (Aggression Questionnaire)

For the total aggression score, a multivariable linear regression was computed with aggression score as the dependent variable and gender, traumatic experiences in childhood, duration of NSSI, frequency of NSSI, pain perception, presence of a suicide attempt, reinforcement mechanisms of NSSI and whether students had received therapy for NSSI as independent variables (Table 2). The regression model was significant (*F*(15,134) = 6.37, *P* < 0.001) and explained 41.60% of variance in the total aggression score. Significant predictors after FDR correction were male gender (*β* = −0.23, *P* = 0.002) and emotional abuse (*β* = 0.51, *P* < 0.001). Regarding the subscales (Supplementary Table 2), physical aggression could only be predicted by male gender. Verbal aggression, anger and hostility and/or distrust could be predicted by more emotional abuse. See Supplementary Table 2 for an overview of regressions regarding the Aggression Questionnaire subscales.

### Linear regression predicting depression (PHQ-9)

For the PHQ-9 score, a multivariable linear regression was computed with PHQ-9 total score as the dependent variable and gender, traumatic experiences in childhood, duration of NSSI, frequency of NSSI, pain perception, presence of a suicide attempt, reinforcement mechanisms of NSSI and whether students had received therapy for NSSI as independent variables (Table 2). The regression model was significant (*F*(15,134) = 3.69, *P* < 0.001) and explained 29.20% of variance in the PHQ-9 score. The only significant predictor after FDR correction was more emotional abuse (*β* = 0.41, *P* < 0.005).

### Binominal logistic regression predicting continuous NSSI

To predict which factors increased the likelihood of continuous NSSI, a binominal logistic regression was computed with NSSI during adolescence or continuous NSSI as the dependent variable and traumatic experiences during childhood, prior therapy, gender, NSSI frequency, pain perception, NSSI reinforcement mechanisms and prior suicide attempts as independent variables (Table 3). NSSI duration was not included (in contrast to the previously computed models), as it is inherently accounted for in the definition of continuous NSSI. The model was significant (χ^2^(14) = 37.26, *P* < 0.001). The model explained 33.00% (Nagelkerke *R* ^2^) of the variance in group assignment and correctly classified 72.90% of cases. More automatic positive reinforcement was the only factor after FDR correction that was associated with continuous NSSI.

**Table 3:** Binominal logistic regression determining the likelihood of factors contributing to continuous non-suicidal self-injury (NSSI)^a^

Dependent variable	Predictors	***B***	s.e.	Wald	**Exp (** ***B*** **)**	**95% CI Exp (** ***B*** **)**	***P***	***R*** **²**
Continuous NSSI	Gender^b^	−0.38	0.70	0.29	0.69	0.17–2.72	0.593	0.33
	Prior therapy	0.42	0.54	0.60	1.52	0.53–4.36	0.437	
	NSSI frequency	−0.47	0.33	1.97	0.63	0.33–1.20	0.161	
	Pain perception	0.02	0.34	0.00	1.02	0.53–1.99	0.948	
	Emotional abuse	0.00	0.06	0.00	1.00	0.90–1.12	0.966	
	Physical abuse	0.17	0.15	1.23	1.18	0.88–1.60	0.267	
	Sexual abuse	0.07	0.08	0.84	1.08	0.92–1.26	0.360	
	Emotional neglect	0.02	0.06	0.07	1.02	0.90–1.15	0.798	
	Physical neglect	−0.17	0.09	3.85	0.84	0.71–1.00	0.050	
	Suicide attempt	1.76	0.93	3.57	5.80	0.94–35.94	0.059	
	ANR	−0.04	0.35	0.01	0.97	0.49–1.90	0.919	
	**APR**	**1.26**	**0.43**	**8.72**	**3.52**	**1.53–8.12**	**0.003**	
	SNR	−0.62	0.55	1.26	0.54	0.18–1.59	0.263	
	SPR	−1.09	0.50	4.68	0.34	0.13–0.90	0.031	

ANR, automatic negative reinforcement; APR, automatic positive reinforcement; SNR, social negative reinforcement; SPR, social positive reinforcement.

a. Significant predictors after FDR correction are marked in bold.

b. Female was coded as 1 and male as 0.

## Discussion

The objective of this study was to determine the relationships among NSSI, traumatic experiences in childhood, and emotional or behavioural symptoms (i.e. depression, aggression and emotional reactivity) in university students. In addition, we investigated which factors increased or reduced the likelihood of NSSI continuing into adulthood instead of solely occurring in adolescence.

Participants who continuously engaged in NSSI did so for longer periods and with a higher maximal frequency during adolescence compared with participants that engaged in NSSI solely during adolescence. Notably, age at onset has previously been previously linked to more frequent NSSI engagement, suggesting that the effectiveness of NSSI may diminish over time, necessitating increased frequency or longer engagement for the same desired effect.^48^ The present study also found that participants with continuous NSSI were more likely to engage in NSSI for automatic positive reinforcement, i.e. in an attempt to regulate their emotions. Automatic types of reinforcement are reported most frequently among individuals engaging in NSSI;^6^ however, differences in reinforcement mechanisms between adolescents and adults have not been investigated in detail. Automatic positive reinforcement may be less likely to lose relevance with increasing age, as it is believed to contribute to the general maintenance of NSSI.^49^ Finally, although the two groups did not differ significantly in their experience with prior therapy, those who had engaged in NSSI continuously had significantly more frequent suicide attempts compared with those who had engaged in NSSI only during adolescence. NSSI is often considered to be a gateway to suicidal ideation and behaviour, even when the self-injury is not intended to be suicidal.^50^ However, a study in adults with borderline personality disorder using real-time monitoring found that NSSI could mitigate suicidal ideation, suggesting that it may serve as a coping strategy to alleviate suicidal thoughts.^51^ This implies that suicidal ideation frequently accompanies NSSI, which might function as either a coping mechanism or a reinforcement of suicidal tendencies, depending on the severity of the self-harm. In individuals who engage in NSSI continuously, suicidal ideation may be more persistent, leading to more frequent suicide attempts and a heightened need to engage in NSSI.

All three domains under examination (depression, aggression and emotional reactivity) shared a key predictor: childhood emotional abuse; these are discussed in detail below. The prevalence of emotional abuse in our sample underscores the importance of early screening during adolescence and addressing emotional abuse in therapy. Future research should focus on how emotional abuse affects various domains of mental health and explore effective strategies for early detection and intervention to mitigate its long-term impacts.

### Predictors of emotional reactivity

Longer NSSI duration and more emotional abuse were significant predictors of overall emotional reactivity. Regarding the subscales, prior therapy and emotional abuse were significant predictors of intensity of the evoked emotions. These results suggest that engaging in NSSI as an emotion regulation strategy for longer periods of time may reinforce general emotional reactivity. However, it is important to acknowledge the cross-sectional design of the study and the possible bidirectional nature of this relationship, as participants’ emotional reactivity before the survey was unknown. Longer NSSI duration could be both a cause and a consequence of increased intensity. This implies the development of a potential vicious cycle, wherein individuals with extended NSSI durations reinforce this strategy for emotion regulation, amplifying their response to heightened intensity. Simultaneously, heightened intensity may drive the need for longer NSSI durations to regulate emotions. Further investigation of this relationship, particularly using longitudinal designs, would be needed to be able to infer directionality of effects.

In addition, prior therapy for NSSI emerged as a contributor to the intensity subscale. This relationship could be bidirectional, as an increased sensitivity to emotional stimuli could lead to crises and, subsequently, the need for treatment. A study by Hamza et al on the relationships among stressful events, emotional reactivity and subsequent NSSI in university students found that emotional reactivity mediated the relationship between stressful events and NSSI.^23^ This suggests that heightened emotional reactivity could drive individuals to use NSSI as a means of emotion regulation, and, as difficulties with emotion regulation and frequent NSSI persist, the likelihood of seeking therapy increases. Emotional abuse – specifically, experiences such as insults and being told one should have never been born – during childhood or adolescence predicted higher general emotional reactivity and intensity. This is in line with previous work linking traumatic experiences to emotional reactivity.^8,23^ A significant contribution of our study is its identification of emotional abuse as the exclusive predictor of emotional reactivity among traumatic experiences. Emotional abuse, often unseen, is posited to have more profound effects than physical and sexual abuse.^52^ Its impact on self-esteem and potential neurobiological alterations to the stress response system^53^ may contribute to the development and maintenance of NSSI.^18,54^ Future research should focus on differentiating specific types of emotional abuse and their relative impacts on emotional reactivity. Investigating how different forms of verbal insults and negative messages contribute to emotional distress could enhance our understanding of the mechanisms at play. In addition, longitudinal studies are needed to track the long-term effects of emotional abuse on emotion regulation and NSSI. This comprehensive approach could ultimately lead to more effective, targeted interventions for individuals affected by emotional abuse.

In summary, although the cross-sectional nature of our analyses limited our ability to make causal inferences, the findings suggest that emotional reactivity is closely associated with NSSI and is particularly influenced by experiences of emotional abuse during childhood. Emotional abuse seems to have a significant role in shaping emotional reactivity, which in turn may be linked to NSSI. Future longitudinal research is needed to better understand the directionality of these effects and the causal relationships between these factors.

### Predictors of aggression

In our study, male gender and higher incidence of emotional abuse emerged as significant predictors of aggression. The literature supports the notion that externalising problems, including aggression, are more prevalent in males.^55^ Although men and women exhibit equal levels of verbal aggression, physical aggression tends to be more prevalent among males.^56^ In our sample, male gender emerged as the sole significant predictor of physical aggression.

Emotional abuse was a predictor of overall aggression and the exclusive predictor of expressions of verbal aggression, anger, and hostility and/or distrust. Previous research has established connections between parental abuse and subsequent hostility in offspring,^57^ with emotional abuse specifically linked to problematic behaviours in adolescents, mediated by heightened levels of anger.^58^ Furthermore, emotional abuse and neglect have been shown to adversely affect emotion regulation skills, which are fundamental to managing aggression, in university students.^59^ Such impaired emotion regulation can increase the likelihood of using NSSI as a strategy for managing intense emotions.^60^

In summary, although our results indicate that aggression is associated with negative childhood experiences, especially emotional abuse, the cross-sectional design limits causal conclusions. The findings suggest connections among emotional abuse, heightened aggression and the use of NSSI. However, longitudinal research is needed to clarify the causal relationships among these factors and the directionality of their effects.

### Predictors of depressive symptoms

Similar to emotional reactivity and aggression, depression scores was mostly influenced by increased emotional abuse. Emotional abuse and neglect are frequently linked to negative mental health outcomes, including depressive disorders.^61,62^ One hypothesis is that emotional abuse undermines children’s resilience to adverse events, leading to increased depressive symptomatology.^63^ Longitudinal studies support the connection between emotional neglect and depressive symptoms, noting, however, the potential protective effect of peer social support.^64^ Thus, emotional neglect may have more severe and detrimental effects when adolescents also experience social exclusion and isolation. Individuals with NSSI frequently report experiences of bullying and isolation,^65^ which may exacerbate the frequency and severity of depressive symptoms. Future research should investigate how emotional abuse affects resilience and coping strategies across different developmental stages, to identify potential interventions. In addition, exploring the dynamics between social support systems and emotional neglect could provide valuable insights into effective preventive measures and therapeutic approaches for mitigating depressive symptoms in populations engaging in NSSI.

### Predictors of continuous NSSI in university students

The final objective of our study was to examine predictors of continuous NSSI compared with NSSI solely in adolescence. We found that increased automatic positive reinforcement was a significant predictor of continuous NSSI. These findings are in line with previous reports^30^ and suggest that individuals persistently involved in NSSI are more likely to view it as an emotion regulation strategy, as they have experienced the efficacy of NSSI in enduring and terminating negative emotional states, thereby reinforcing NSSI. Without alternative (and similarly effective) emotion regulation strategies, individuals may have no reason to refrain from NSSI, even after adolescence. Notably, although emotional abuse was a significant predictor of emotional reactivity, aggression and depressive symptoms, it did not emerge as a significant predictor of continuous NSSI. One possible explanation is that emotional abuse is a common experience among both individuals with intermittent NSSI and those with continuous NSSI and is thus a general risk factor rather than a specific predictor of NSSI persistence. Further research is needed to explore these dynamics and clarify the role of emotional abuse in the persistence of NSSI. Overall, these results highlight the importance of introducing alternative emotion regulation strategies, particularly during adolescence, to mitigate the risk of continuous self-harm in adulthood. In addition, it is crucial to investigate underlying issues related to emotion regulation, such as potential difficulties with alexithymia,^66^ which some research suggests may have a role in emotional dysregulation and self-harm behaviours. Understanding these aspects could help us to tailor more effective interventions that address both the emotional and regulatory challenges that individuals face.

### Limitations and strengths of the study

The present study consisted of a sample of university students from various fields of study and study programmes. Therefore, the results may not be generalisable to other populations, and separate studies will be needed for diverse groups. Overall, a high level of motivation and honesty on the part of the participants can be assumed owing to the low remuneration (raffle of 25 vouchers worth 15 euros) and anonymous participation. Nevertheless, there was a high drop-out rate; 240 people clicked on the questionnaire, yet only 150 were finally included in the analysis. There are various potential reasons for this, e.g. emotional stress caused by the topics addressed, the duration of the survey and fulfilment of exclusion criteria. However, according to our power analysis, enough participants were included. An additional limitation is the cross-sectional nature of our online survey and its reliance on self-reported data with retrospective queries for traumatic experiences and NSSI characteristics. To explore causal relationships, future studies should adopt a longitudinal design, possibly complemented by third-party-reported data and detailed assessment of possible psychiatric diagnoses for enhanced accuracy and reliability. Assessment of externalising disorders (e.g. attention-deficit hyperactivity disorder, substance misuse) will be particularly important owing to the strong correlations of these disorders with aggression and emotion regulation. Furthermore, although we used well-validated questionnaires, it is important to consider the reduced reliability of the CTQ physical neglect scale. Finally, the study population consisted of young adults, and it is likely that their engagement in NSSI will vary and evolve over time. Future longitudinal research should examine how NSSI behaviours develop, change and stabilise over the lifespan, comparing individuals who engage in NSSI exclusively during adolescence, those who continue these behaviours into adulthood, and control groups with no history of NSSI. Understanding these dynamics could provide valuable insight into the progression and persistence of NSSI and inform targeted interventions across different life stages.

### Outlook

Future research should aim to replicate the associations reported here and investigate the directionality of the links between emotional reactivity and NSSI. Last, intervention studies are warranted to assess the efficacy of alternative emotion regulation strategies, specifically targeting individuals who predominantly resort to NSSI for emotion regulation.

## Supporting information

Jarvers et al. supplementary material 1 Jarvers et al. supplementary material

Jarvers et al. supplementary material 2 Jarvers et al. supplementary material

## Data Availability

The data that support the findings of this study are available from the corresponding author, D.S., upon reasonable request.
