# Supplementary material for: Characteristics of self-injurious behaviour and early traumatic experiences: associations with emotional reactivity, depression and aggression in university students
Source: BJPsych Open. 2025 Mar 11;11(2):e45. doi: 10.1192/bjo.2024.862 (PMC12001921; doi:10.1192/bjo.2024.862)
Supplement: Jarvers et al. supplementary material 1 — Jarvers et al. supplementary material [file S2056472424008627sup001.docx]

| **Table S1**  Overview of linear regressions predicting the ERS subscales sensitivity, intensity/arousal and persistence. | | | | | | | | |  |
| --- | --- | --- | --- | --- | --- | --- | --- | --- | --- |
| **Dependent Variable** | **Predictors** | ***B*** | **SE** | **95 % KI *B*** | **β** | ***t*** | ***p*** | ***R²*** | |
| Sensitivity | Gender* | 1.33 | 2.01 | -2.64 – 5.31 | .06 | 0.66 | .508 | .23 | |
|  | Prior therapy | 2.79 | 1.50 | -0.18 – 5.77 | .16 | 1.86 | .065 |  | |
|  | NSSI duration | 1.42 | 0.70 | 0.04 – 2.81 | .18 | 2.03 | .044 |  | |
|  | NSSI frequency | -1.79 | 1.29 | -4.35 – 0.77 | -.13 | -1.38 | .169 |  | |
|  | Pain perception | -0.15 | 0.98 | -2.09 – 1.79 | -.01 | -0.15 | .879 |  | |
|  | Emotional abuse | 0.50 | 0.18 | 0.14 – 0.86 | .41 | 2.77 | .006 |  | |
|  | Physical abuse | -0.60 | 0.35 | 0.09 – 0.58 | .35 | -1.73 | .086 |  | |
|  | Sexual abuse | -0.27 | 0.19 | -0.65 – 0.10 | -.12 | -1.46 | .147 |  | |
|  | Emotional neglect | -0.11 | 0.20 | -0.50 – 0.28 | -.06 | -0.55 | .583 |  | |
|  | Physical neglect | -0.20 | 0.27 | -0.73 – 0.34 | -.08 | -0.72 | .476 |  | |
|  | Suicide attempt | 2.56 | 1.97 | -1.34 – 6.46 | .12 | 1.30 | .196 |  | |
|  | ANR | -0.32 | 1.07 | -2.43 – 1.80 | -.03 | -0.30 | .767 |  | |
|  | APR | 1.51 | 1.19 | -0.83 – 3.86 | .14 | 1.28 | .204 |  | |
|  | SNR | 2.41 | 1.72 | -0.99 – 5.80 | .11 | 1.40 | .163 |  | |
|  | SPR | 2.40 | 1.61 | -0.78 – 5.57 | .13 | 1.40 | .138 |  | |
| Intensity/ Arousal | Gender* | 0.07 | 1.55 | -3.00 – 3.14 | .01 | 0.05 | .964 | .26 | |
|  | **Prior therapy** | **3.33** | **1.16** | **1.03 – 5.62** | **.24** | **2.87** | **.005** |  | |
|  | NSSI duration | 1.27 | 0.54 | 0.20 – 2.34 | .21 | 2.35 | .020 |  | |
|  | NSSI frequency | -0.67 | 0.99 | -2.65 – 1.30 | -.06 | -0.67 | .502 |  | |
|  | Pain perception | -0.56 | 0.76 | -2.06 – 0.94 | -.06 | -0.74 | .462 |  | |
|  | **Emotional abuse** | **0.44** | **0.14** | **0.16 – 0.72** | **.35** | **3.15** | **.002** |  | |
|  | Physical abuse | -0.46 | 0.27 | -0.99 – 0.08 | -.17 | -1.69 | .093 |  | |
|  | Sexual abuse | -0.18 | 0.15 | -0.46 – 0.11 | -.10 | -1.20 | .231 |  | |
|  | Emotional neglect | -0.22 | 0.15 | -0.53 – 0.08 | -.17 | -1.46 | .147 |  | |
|  | Physical neglect | -0.08 | 0.21 | -0.50 – 0.34 | -.04 | -0.38 | .707 |  | |
|  | Suicide attempt | 1.79 | 1.52 | -1.22 – 4.80 | .10 | 1.17 | .242 |  | |
|  | ANR | 0.13 | 0.83 | -1.50 – 1.76 | .02 | 0.16 | .876 |  | |
|  | APR | 0.74 | 0.92 | -1.07 – 2.55 | .09 | 0.81 | .422 |  | |
|  | SNR | 1.02 | 1.33 | -1.60 – 2.64 | .06 | 0.77 | .443 |  | |
|  | SPR | 1.79 | 1.24 | -0.66 – 4.24 | .12 | 1.44 | .151 |  | |
| Persistence | Gender* | 0.94 | 0.89 | -0.83 – 2.70 | .09 | 1.05 | .296 | .19 | |
|  | Prior therapy | 0.66 | 0.67 | -0.66 – 1.97 | .09 | 0.98 | .328 |  | |
|  | NSSI duration | 0.51 | 0.31 | -0.10 – 1.13 | .15 | 1.65 | .101 |  | |
|  | NSSI frequency | -1.43 | 0.57 | -2.56 – -0.29 | -.24 | -2.49 | .014 |  | |
|  | Pain perception | 0.35 | 0.44 | -0.52 – 1.21 | .07 | 0.79 | .430 |  | |
|  | Emotional abuse | 0.15 | 0.08 | -0.01 – 0.31 | .22 | 1.84 | .067 |  | |
|  | Physical abuse | -0.17 | 0.16 | -0.47 – 0.14 | -.11 | -1.09 | .277 |  | |
|  | Sexual abuse | -0.08 | 0.08 | -0.25 – 0.09 | -.08 | -0.96 | .337 |  | |
|  | Emotional neglect | 0.00 | 0.09 | -0.17 – 0.18 | .01 | 0.05 | .963 |  | |
|  | Physical neglect | -0.22 | 0.12 | -0.46 – 0.02 | -.20 | -1.78 | .077 |  | |
|  | Suicide attempt | 1.19 | 0.88 | -0.54 – 2.92 | .13 | 1.36 | .176 |  | |
|  | ANR | 0.200 | 0.475 | -0.74 – 1.14 | .05 | 0.42 | .675 |  | |
|  | APR | 1.018 | 0.526 | -0.02 – 2.06 | .22 | 1.93 | .055 |  | |
|  | SNR | 0.765 | 0.762 | -0.74 – 2.27 | .08 | 1.00 | .318 |  | |
|  | SPR | -0.07 | 0.71 | -1.48 – 1.34 | -.01 | -0.10 | .923 |  | |
|  |  |  |  |  |  |  |  |  | |

*Note.* ERS = Emotion Reactivity Scale; ANR = automatic negative reinforcement; APR = automatic positive reinforcement; SNR = social negative reinforcement; SPR = social positive reinforcement. NSSI = non-suicidal self-injury. Significant predictors after FDR-correction are marked in bold font. *female was coded as 1 and male as 0
