# Supplementary material for: Characteristics of self-injurious behaviour and early traumatic experiences: associations with emotional reactivity, depression and aggression in university students
Source: BJPsych Open. 2025 Mar 11;11(2):e45. doi: 10.1192/bjo.2024.862 (PMC12001921; doi:10.1192/bjo.2024.862)
Supplement: Jarvers et al. supplementary material 2 — Jarvers et al. supplementary material [file S2056472424008627sup002.docx]

| **Table S2**  Overview of linear regressions predicting the AQ subscales physical aggression, verbal aggression hostility/distrust and anger. | | | | | | | | |  |
| --- | --- | --- | --- | --- | --- | --- | --- | --- | --- |
| **Dependent Variable** | **Predictors** | ***B*** | **SE** | **95 % KI *B*** | **β** | ***t*** | ***p*** | ***R²*** | |
| Physical Aggression | **Gender*** | **-4.14** | **0.94** | **-5.99** – **-2.29** | **-.36** | **-4.43** | **<.001** | .31 | |
|  | Prior therapy | -0.07 | 0.70 | -1.45 – 1.31 | -.01 | -0.10 | .918 |  | |
|  | NSSI duration | 0.46 | 0.33 | -0.19 – 1.10 | .12 | 1.40 | .163 |  | |
|  | NSSI frequency | 0.44 | 0.60 | -0.76 – 1.63 | .07 | 0.72 | .471 |  | |
|  | Pain perception | 0.43 | 0.46 | -0.47 – 1.34 | .07 | 0.95 | .344 |  | |
|  | Emotional abuse | 0.08 | 0.08 | -0.09 – 0.25 | .10 | 0.95 | .345 |  | |
|  | Physical abuse | 0.11 | 0.16 | -0.21 – 0.43 | .06 | 0.67 | .506 |  | |
|  | Sexual abuse | -0.13 | 0.09 | -0.30 – 0.05 | -.12 | -1.45 | .149 |  | |
|  | Emotional neglect | -0.07 | 0.09 | -0.25 – 0.12 | -.08 | -0.73 | .469 |  | |
|  | Physical neglect | 0.16 | 0.13 | -0.10 – 0.41 | .13 | 1.23 | .221 |  | |
|  | Suicide attempt | 1.53 | 0.92 | -0.29 – 3.34 | .14 | 1.67 | .098 |  | |
|  | ANR | 0.45 | 0.50 | -0.54 – 1.43 | .10 | 0.89 | .373 |  | |
|  | APR | -0.53 | 0.55 | -1.62 – 0.57 | -.10 | -0.95 | .343 |  | |
|  | SNR | 1.32 | 0.80 | -0.26 – 2.90 | .13 | 1.66 | .100 |  | |
|  | SPR | 1.03 | 0.75 | -0.45 – 2.50 | .11 | 1.38 | .171 |  | |
| Verbal Aggression | Gender* | -1.29 | 0.64 | -2.55 – -0.02 | -.17 | -2.00 | .047 | .24 | |
|  | Prior therapy | 0.16 | 0.48 | -0.79 – 1.10 | .03 | 0.32 | .747 |  | |
|  | NSSI duration | 0.19 | 0.22 | -0.25 – 0.63 | .08 | 0.84 | .402 |  | |
|  | NSSI frequency | 0.79 | 0.41 | -0.03 – 1.61 | .18 | 1.91 | .058 |  | |
|  | Pain perception | -0.15 | 0.31 | -0.77 – 0.48 | -.04 | -0.46 | .644 |  | |
|  | **Emotional abuse** | **0.20** | **0.06** | 0.09 – 0.32 | **.39** | **3.45** | **.001** |  | |
|  | Physical abuse | -0.13 | 0.11 | **-0.35 – 0.09** | -.12 | -1.19 | .238 |  | |
|  | Sexual abuse | 0.07 | 0.06 | -0.05 – 0.19 | .10 | 1.17 | .243 |  | |
|  | Emotional neglect | -0.11 | 0.06 | -0.23 – 0.02 | -.19 | -1.67 | .098 |  | |
|  | Physical neglect | 0.18 | 0.09 | 0.01 – 0.36 | .23 | 2.11 | .037 |  | |
|  | Suicide attempt | 0.63 | 0.63 | -0.62 – 1.87 | .09 | 0.99 | .322 |  | |
|  | ANR | 0.21 | 0.34 | -0.46 – 0.89 | .07 | 0.62 | .535 |  | |
|  | APR | -0.98 | 0.38 | -1.73 – -0.23 | -.28 | -2.60 | .011 |  | |
|  | SNR | 0.36 | 0.55 | -0.72 – 1.45 | .05 | 0.66 | .512 |  | |
|  | SPR | 0.40 | 0.51 | -0.62 – 1.41 | .07 | 0.77 | .440 |  | |
| Anger | Gender* | -1.25 | 0.89 | -3.02 – 0.52 | -.12 | -1.40 | .165 | .28 | |
|  | Prior therapy | 1.19 | 0.67 | -0.13 – 2.51 | .15 | 1.79 | .076 |  | |
|  | NSSI duration | 0.16 | 0.31 | -0.46 – 0.78 | .05 | 0.51 | .609 |  | |
|  | NSSI frequency | 0.75 | 0.58 | -0.39 – 1.89 | .12 | 1.31 | .193 |  | |
|  | Pain perception | 0.03 | 0.44 | -0.83 – 0.90 | .01 | 0.08 | .939 |  | |
|  | **Emotional abuse** | **0.37** | **0.08** | **0.21 – 0.53** | **.50** | **4.54** | **<.001** |  | |
|  | Physical abuse | -0.15 | 0.16 | -0.46 – 0.16 | -.10 | -0.98 | .329 |  | |
|  | Sexual abuse | -0.10 | 0.08 | -0.26 – 0.07 | -.09 | -1.16 | .248 |  | |
|  | Emotional neglect | -0.13 | 0.09 | -0.30 – 0.05 | -.16 | -1.41 | .160 |  | |
|  | Physical neglect | -0.03 | 0.12 | -0.27 – 0.22 | -.02 | -0.21 | .835 |  | |
|  | Suicide attempt | 0.66 | 0.88 | -1.08 – 2.40 | .07 | 0.75 | .455 |  | |
|  | ANR | 0.14 | 0.48 | -0.80 – 1.08 | .03 | 0.30 | .767 |  | |
|  | APR | 0.00 | 0.53 | -1.04 – 1.04 | .00 | -0.00 | .999 |  | |
|  | SNR | 1.05 | 0.76 | -0.46 – 2.56 | .11 | 1.38 | .171 |  | |
|  | SPR | 0.68 | 0.71 | -0.73 – 2.09 | .08 | 0.96 | .342 |  | |
| Hostility/  Distrust | Gender* | -0.92 | 1.13 | -2.15 – 1.32 | -.06 | -0.81 | .418 | .34 | |
|  | Prior therapy | 0.04 | 0.84 | -1.63 – 1.70 | .00 | 0.05 | .965 |  | |
|  | NSSI duration | -0.42 | 0.39 | -1.19 – 0.36 | -.09 | -1.06 | .293 |  | |
|  | NSSI frequency | 0.21 | 0.73 | -1.22 – 1.65 | .03 | 0.29 | .769 |  | |
|  | Pain perception | -0.10 | 0.55 | -1.19 – 0.99 | -.01 | -0.19 | .853 |  | |
|  | **Emotional abuse** | **0.50** | **0.10** | **0.30 – 0.70** | **.51** | **4.88** | **<.001** |  | |
|  | Physical abuse | -0.18 | 0.20 | -0.56 – 0.21 | -.08 | -0.89 | .374 |  | |
|  | Sexual abuse | -0.10 | 0.11 | -0.31 – 0.11 | -.07 | -0.92 | .361 |  | |
|  | Emotional neglect | 0.03 | 0.11 | -0.20 – 0.25 | .02 | 0.22 | .825 |  | |
|  | Physical neglect | 0.11 | 0.15 | -0.20 – 0.41 | .07 | 0.70 | .484 |  | |
|  | Suicide attempt | 1.47 | 1.11 | -0.72 – 3.65 | .11 | 1.33 | .187 |  | |
|  | ANR | -0.41 | 0.60 | -1.59 – 0.78 | -.07 | -0.68 | .500 |  | |
|  | APR | 0.48 | 0.67 | -0.83 – 1.80 | .07 | 0.73 | .469 |  | |
|  | SNR | 1.25 | 0.96 | -0.66 – 3.15 | .10 | 1.30 | .198 |  | |
|  | SPR | 0.46 | 0.90 | -1.32 – 2.24 | .04 | 0.51 | .611 |  | |
|  |  |  |  |  |  |  |  |  | |

*Note.* AQ = Aggression Questionnaire; ANR = automatic negative reinforcement; APR = automatic positive reinforcement; SNR = social negative reinforcement; SPR = social positive reinforcement. NSSI = non-suicidal self-injury. Significant predictors after FDR-correction are marked in bold font. *female was coded as 1 and male as 0
